# Antiangiogenic and Neurogenic Activities of* Sleeping Beauty*-Mediated PEDF-Transfected RPE Cells* In Vitro* and* In Vivo*

**DOI:** 10.1155/2015/863845

**Published:** 2015-12-01

**Authors:** Sandra Johnen, Yassin Djalali-Talab, Olga Kazanskaya, Theresa Möller, Nina Harmening, Martina Kropp, Zsuzsanna Izsvák, Peter Walter, Gabriele Thumann

**Affiliations:** ^1^Department of Ophthalmology, University Hospital RWTH Aachen, Pauwelsstraße 30, 52074 Aachen, Germany; ^2^Department of Ophthalmology, University Hospitals of Geneva, Street Alcide-Jentzer 22, 1211 Geneva 14, Switzerland; ^3^Max Delbrück Center for Molecular Medicine in the Helmholtz Association, Robert-Rössle-Straße 10, 13125 Berlin, Germany

## Abstract

Pigment epithelium-derived factor (PEDF) is a potent multifunctional protein that inhibits angiogenesis and has neurogenic and neuroprotective properties. Since the wet form of age-related macular degeneration is characterized by choroidal neovascularization (CNV), PEDF would be an ideal candidate to inhibit CNV and support retinal pigment epithelial (RPE) cells. However, its short half-life has precluded its clinical use. To deliver PEDF to the subretinal space, we transfected RPE cells with the* PEDF* gene using the* Sleeping Beauty* transposon system. Transfected cells expressed and secreted biologically active recombinant PEDF (rPEDF). In cultures of human umbilical vein endothelial cells, rPEDF reduced VEGF-induced cumulative sprouting by ≥47%, decreased migration by 77%, and increased rate of apoptosis at least 3.4 times. rPEDF induced neurite outgrowth in neuroblastoma cells and protected ganglion and photoreceptor cells in organotypic retinal cultures. In a rat model of CNV, subretinal transplantation of PEDF-transfected cells led to a reduction of the CNV area by 48% 14 days after transplantation and decreased clinical significant lesions by 55% and 40% after 7 and 14 days, respectively. We showed that transplantation of pigment epithelial cells overexpressing* PEDF* can restore a permissive subretinal environment for RPE and photoreceptor maintenance, while inhibiting choroidal blood vessel growth.

## 1. Introduction

The avascularity of the outer retina is essential to maintain a proper neurogenic environment for the photoreceptors and retinal neurons, which could be harmed by changes in the composition of blood, such as an excess of glutamate [1]. Retinal pigment epithelial (RPE) cells form a tight monolayer at the interface between the neural retina and the choroid and prevent the choroidal vasculature from invading the subretinal space and the outer retina. RPE cells are important in maintaining the angiogenic homeostasis of the outer retina by synthesizing and secreting angioregulatory proteins, including the proangiogenic vascular endothelial growth factor (VEGF) [2, 3] and the antiangiogenic pigment epithelium-derived factor (PEDF) [4–6]. Even though other factors, such as fibroblast growth factor and transforming growth factor-beta [7], endostatin [8, 9], and thrombospondin-1 [6, 10], contribute, it is the equilibrium between VEGF and PEDF that is most responsible for maintaining the outer retina avascular [11, 12]. It is the imbalance in proangiogenic and antiangiogenic factors that has been implicated in a number of severe ocular diseases, such as neovascular age-related macular degeneration (AMD), diabetic retinopathy, sickle cell retinopathy, and retinopathy of prematurity [13–16]. Even though neovascularization is a complex event that requires endothelial cell proliferation, migration, and tissue degradation, it has been assumed that inhibition of VEGF is sufficient to inhibit choroidal neovascularization (CNV). Based on this assumption, inhibitors of VEGF have been developed and successfully used to control neovascularization under pathological conditions, such as cancer [17], macular edema [18], retinopathy of prematurity [19], and neovascular AMD [20, 21].

The first VEGF inhibitor approved for ocular use, specifically for the treatment of neovascular AMD, was pegaptanib, a synthetic PEGylated oligonucleotide that binds the VEGF-A_165_ isoform. However, its effectiveness as a treatment of neovascular AMD was minimal [22]. In 2006, the Food and Drug Administration (FDA) approved ranibizumab (Lucentis, Novartis Pharma GmbH), a humanized Fab fragment derived from the parent monoclonal antibody bevacizumab (Avastin, Roche), for intraocular use to treat neovascular AMD. Intravitreal injection of ranibizumab controls CNV in 90% of patients with significant vision improvement in 30–40% of patients [23, 24]. In addition to ranibizumab, the parent monoclonal antibody bevacizumab and the recombinant fusion protein aflibercept (Eylea, Bayer HealthCare) are also used for the treatment of neovascular AMD [20, 21]. Since the intravitreal half-life of these drugs is short, patients need frequent, often monthly injections for life to reduce the leakage from CNV. The logistic of the frequent intravitreal injections, especially in the older patient, often results in the patient discontinuing treatment. Intravitreal injections have also been linked to local side effects, such as endophthalmitis, ocular hypertension [25], submacular hemorrhage [26], and rarely occurring thromboembolic events [27].

The replacement of damaged RPE by subretinal transplantation of autologous RPE or iris pigment epithelial (IPE) cells in neovascular AMD patients [28, 29] has not shown any significant improvement in functional outcome. We hypothesize that subretinal transplantation of pigment epithelial cells that secrete therapeutic levels of PEDF continuously for the life of the patient will inhibit and induce regression of CNV without local and systemic side effects. In fact, a number of investigators have reported that the subconjunctival injection of recombinant PEDF (rPEDF) [30] and the intravitreal or subretinal administration of viral vectors encoding the* PEDF* gene control ocular neovascularization [31, 32]. However, injection of PEDF protein to control CNV is not feasible since it has a short half-life [33] and would require frequent injections. Administration of viral vectors encoding the* PEDF* gene is an option to control CNV, although viral vectors may have a number of limitations that preclude their clinical use, mostly related to their immunogenicity [34–36]. Ocular gene therapies using adenoviral and adenoassociated viral vectors have been used in clinical trials with success. However, some patients have shown mild to moderate local as well as systemic adverse-event profiles [37–39]. In 2006, phase I clinical trial including patients with advanced neovascular AMD showed that high doses of intravitreally injected adenoviral vectors expressing human PEDF led to antiangiogenic activity lasting several months [40]. However, no follow-up clinical trial has been reported.

To avoid the side effects associated with virally mediated gene delivery, we have used the hyperactive* Sleeping Beauty* (*SB100X*) transposon system to transfect pigment epithelial cells with the* PEDF* gene [41].* SB100X* has a number of advantages over virally mediated gene delivery: it integrates transgenes exclusively into TA dinucleotides with specific predilection for dinucleotides that have distinct structural features, integrates transgenes with high efficiency, results in stable integration, integrates large inserts, and does not primarily integrate into transcriptional sequences [42–45].

This study was designed to investigate whether the* SB100X* transposon system can efficiently transfect primary pigment epithelial cells with the human* PEDF* gene and whether the secreted rPEDF is functional as an inhibitor of VEGF-mediated endothelial cell function and as a neuroprotective agent* in vitro* and whether PEDF-transfected pigment epithelial cells have the potential to inhibit neovascularization* in vivo* in a model of CNV.

## 2. Materials and Methods

### 2.1. Cell Culture

Human umbilical vein endothelial cells (HUVEC) were isolated as described by Korff and Augustin [46] and cultured in endothelial cell growth (ECG) medium, containing 0.02 mL/mL fetal calf serum, 0.004 mL/mL endothelial cell growth supplement, 0.1 ng/mL recombinant human epidermal growth factor, 1 ng/mL recombinant human basic fibroblast growth factor, 90 *μ*g/mL heparin, and 1 *μ*g/mL hydrocortisone (PromoCell, Heidelberg, Germany), and supplemented with 80 U/mL penicillin, 80 *μ*g/mL streptomycin (Lonza, Basel, Switzerland), and 2.5 *μ*g/mL amphotericin B (Sigma-Aldrich Chemie, Taufkirchen, Germany). Rat RPE cells were isolated from Long Evans rats as described previously [41]. Briefly, eyes were cut circumferentially below the ora serrata, the neural retina was removed, and the eye cup was filled with 0.05% trypsin (PAA Laboratories, Pasching, Austria). After 10 minutes, the RPE cells were dislodged by trituration. An equal volume of Dulbecco's Modified Eagle's Medium (DMEM)/Ham's F-12 (Biochrom AG, Berlin, Germany) supplemented with 10% fetal bovine serum (FBS, PAA Laboratories) was added to the trypsin-cell mixture and centrifuged at 1000 rpm for 5 minutes. The RPE cell pellet was suspended in DMEM/Ham's F-12 supplemented with 10% FBS, 80 U/mL penicillin, 80 *μ*g/mL streptomycin, and 2.5 *μ*g/mL amphotericin B and plated into tissue culture dishes. Second passage cells were immortalized by transfection with pSV3-neo plasmid. Immortalized cells were maintained in DMEM/Ham's-F-12 supplemented with 10% FBS, 80 U/mL penicillin, 80 *μ*g/mL streptomycin, and 2.5 *μ*g/mL amphotericin B at 37°C in a humidified atmosphere of 95% air and 5% CO_2_. Medium was changed twice a week and cells were passaged every 10 days. ARPE-19 cells (ATCC CRL-2302) were maintained and passaged weekly according to the depositors recommendations.

### 2.2. Culture and Transfection of RPE Cells with the* PEDF* Gene

Construction of the PEDF transposon plasmid, whose expression cassette contains the His-tag fused human* PEDF* gene flanked by inverted repeat/direct repeat regions, and transfection using the* SB100X* transposon system have been reported previously [41]. Briefly, RPE cells were electroporated with the Neon Transfection System using the 10 *μ*L Kit (Invitrogen, Carlsbad, CA). 10^5^ cells in 11 *μ*L resuspension buffer R (Invitrogen) were added to 0.5 *μ*g plasmid mixture, containing the* SB100X* transposase plasmid and the PEDF transposon plasmid at a ratio of 1 : 16. Electroporation was carried out using 1350 V (pulse voltage), 20 ms (pulse width), and 2 pulses. After transfection, ARPE-19 and immortalized rat RPE cells were transferred into 12-well tissue culture plates in 2 mL DMEM/Ham's F-12 media supplemented with 10% FBS without antibiotics. Antibiotics were added with the first medium change 3 days after transfection when the transfected cells were transferred into 75 cm^2^ cell culture flasks and the media were analyzed for rPEDF secretion. Established rat RPE and transfected ARPE-19 cells were passaged weekly.

### 2.3. Purification and Analysis of rPEDF Secreted into Culture Media

Recombinant PEDF was purified using Ni-NTA resin (Qiagen, Hilden, Germany) according to the manufacturer's instructions. Briefly, cell culture media from PEDF-transfected cells were collected, clarified by centrifugation for 10 minutes at 1000 rpm at 4°C, mixed with one-third volume of 4x incubation buffer (200 mM NaH_2_PO_4_, pH 8.0, 1.2 M NaCl, and 40 mM imidazole), and applied to Ni-NTA resin. After washing the resin twice with 1x incubation buffer, the bound protein was eluted with the appropriate volume of elution buffer (50 mM NaH_2_PO_4_, pH 8.0, 300 mM NaCl, and 250 mM imidazole). Purified rPEDF was equilibrated with PBS, concentrated on Microcon columns (Merck Millipore, Billerica, MA), sterile-filtered, and stored in aliquots at 4°C until use.

### 2.4. Quantification of rPEDF in Rat Eyes

Quantification of rPEDF in rat eyes after cell transplantation was carried out by homogenization of the whole eye and incubation of the homogenate with 100 *μ*L of radioimmunoprecipitation assay (RIPA) buffer (Pierce Biotechnology, Rockford, IL). RIPA-extracted homogenates were separated on a 10% SDS-polyacrylamide gel. For* Western* blot analysis, proteins were transferred onto a 0.45 *μ*m pore size nitrocellulose membrane (Whatman, Maidstone, Kent, UK). Blots, blocked with 3% BSA/TRIS-buffered saline (TBS) overnight at 4°C, were incubated with anti-PEDF antibodies (rabbit polyclonal, 1 : 4000; BioProducts MD, Middletown, MD) diluted in 3% BSA/TBS at room temperature. After 2 hours, blots were incubated for 1 hour at room temperature with horseradish peroxidase-conjugated anti-rabbit antibodies (goat polyclonal, 1 : 2000; Abcam, Cambridge, United Kingdom) diluted in 10% milk powder/TBS. Protein bands were visualized by chemiluminescence using the LAS-3000 imaging system (FujiFilm, Tokyo, Japan). ELISA-based quantification was performed using the ELISAquant kit for human PEDF (BioProducts MD) and Ni-NTA HisSorb plates (Qiagen) according to the manufacturer's instructions.

### 2.5. Neurite Outgrowth

Human neuroblastoma SH-SY5Y cells (CLS Cell Lines Service GmbH, Eppelheim, Germany) were used to examine the effects of rPEDF on neurite outgrowth. SH-SY5Y cells were seeded in 24-well culture plates at a density of 5 × 10^3^ cells/cm^2^. After the cells were adapted to serum-free DMEM/Ham's F-12 for 24 hours, affinity-purified rPEDF was added at 20 and 100 ng/mL for a period of 5 days. Control cells were maintained in serum-free medium alone. Neurite outgrowth was visualized in living cultures by phase contrast microscopy. For each treatment, 6 microscopic fields were imaged and the total neurite length was measured using the ImageJ software (National Institutes of Health, Bethesda, MD) [47]. Total neurite length was related to the total area of cell bodies as a parameter proportional to cell number.

### 2.6. Organotypic Retinal Culture

Cultures of bovine neural retinas were prepared as previously described [48, 49] and cultivated in the absence or presence of 100 ng/mL purified rPEDF for a period of 72 hours. Tissues were analyzed by hematoxylin/eosin staining or processed for immunohistochemistry and compared to sections of freshly isolated retinas. Staining of photoreceptor cell outer segments was carried out with anti-rhodopsin antibodies (mouse monoclonal, clone 1D4, 1 : 500; Sigma-Aldrich, Taufkirchen, Germany); nuclear staining was performed using DAPI (Roche Applied Science, Mannheim, Germany).

### 2.7. Spheroid-Based Angiogenesis Assay

Endothelial cell spheroids of defined cell numbers were generated as described by Korff and Augustin [46]. Briefly, HUVEC were suspended in ECG medium containing 20% (w/v) carboxymethyl cellulose (Sigma-Aldrich) and seeded in 25 *μ*L drops containing 400 cells on nonadherent plastic Petri dishes (Greiner Bio-One GmbH, Frickenhausen, Germany). Under these conditions, all suspended cells contribute to the formation of a single spheroid per drop. After overnight incubation at 37°C in a humidified atmosphere of 95% air and 5% CO_2_, the spheroids were embedded into collagen gels and rapidly transferred into prewarmed 24-well tissue culture plates. After 30 minutes at 37°C to allow the collagen to polymerize, 100 *μ*L of endothelial basal medium, medium containing 20 ng/mL VEGF with and without the addition of purified rPEDF at concentrations of 20, 100, and 200 ng/mL, and bevacizumab at concentrations of 50, 250, and 500 *μ*g/mL were layered on top of the gels. After 24 hours, capillary sprouting was quantified by measuring the cumulative length of sprouts using the digital imaging software ImageJ.

### 2.8. TUNEL Assay

The effect of VEGF, rPEDF, and bevacizumab on HUVEC apoptosis was analyzed using TUNEL staining (In Situ Cell Death Detection Kit, Fluorescein, Roche Applied Science) according to the manufacturer's protocol.

### 2.9. Migration Assay

HUVEC were plated in complete ECG medium in the upper chamber of an 8 *μ*m pore size transwell insert. After 4 hours at 37°C to allow the cells to attach, the media were replaced with minimal ECG medium supplemented with 2% FBS, with and without the addition of purified rPEDF and bevacizumab at concentrations of 100 ng/mL and 250 *μ*g/mL, respectively. The lower chamber was filled with minimal ECG medium supplemented with 2% FBS, with and without the addition of 50 ng/mL VEGF. After 8 hours at 37°C in a humidified atmosphere of 95% air and 5% CO_2_, the membranes were fixed in formalin and stained with DAPI. Cells remaining on the upper side of the filter were removed by gently wiping with a cotton swab and allowing the filter to air dry.

### 2.10. Animal Experiments

All animal experiments were performed in accordance with the ARVO declaration for the use of animals in ophthalmic research and in accordance with the German Law for the Protection of Animals, after approval was obtained by the regulatory authorities. Rats were anesthetized by an intraperitoneal injection of a mixture of ketamine (75 mg/kg; Ceva Tiergesundheit GmbH, Düsseldorf, Germany) and xylazine (10 mg/kg; Medistar Arzneimittelvertrieb GmbH, Ascheberg, Germany). 32 male, 8-week-old Long Evans rats, weighing approximately 300 g, were used for quantification of rPEDF after injection. They were divided into two groups: one was transplanted with* Venus*-transfected RPE cells and one was transplanted with PEDF-transfected rat RPE cells.

### 2.11. Laser-Induced CNV

CNV was generated by laser-induced rupture of Bruch's membrane in 40 male, 8-week-old Long Evans rats, weighing approximately 300 g. Briefly, rats were anesthetized and pupils were dilated with phenylephrine hydrochloride (2.5%) and tropicamide (0.5%). With a coverslip used as a contact lens, 6 argon laser spots (150 mW intensity, 100 ms duration, and 50 *μ*m size; Carl Zeiss Meditec AG, Jena, Germany) were delivered to the retina and choroid of the right eye of each rat. Production of a bubble at the time of laser exposure as indication of correct focusing was noted and only these laser spots were included in the study. To investigate the effect of cell transplantation on laser-induced CNV, the laser-treated rats were randomized into 4 groups of 10 rats each: one group was transplanted subretinally with PEDF-transfected rat RPE cells, one group was transplanted with* Venus*-transfected RPE cells, one group was injected subretinally with a volume of PBS equal to the volume of cells, and one group served as the noninjected control. Eight additional rats were used for choroidal flat-mount preparations 8 weeks after laser-induced CNV. Transfected RPE cells were suspended in PBS at a density of 5 × 10^3^ cells/*μ*L; 2 *μ*L was injected into the subretinal space of the right eye between the host RPE and photoreceptor cell layers, using a de Juan cannula (Bausch + Lomb, Rochester, NY) attached to a 10 *μ*L Hamilton syringe (Hamilton Messtechnik GmbH, Höchst, Germany). Prior to cell transplantation, a channel leading to the subretinal space was generated using a 30-gauge cannula. Sham injections containing an equivalent volume of PBS were injected into the dorsal subretinal space in an identical manner.

### 2.12. Fluorescein Angiography

Fluorescein angiography was performed in anesthetized rats at days 7 and 14 after laser photocoagulation using the Retina Angiograph II imaging system (Heidelberg Engineering GmbH, Heidelberg, Germany). After intraperitoneal injection of 1 mL of 2.5% sodium fluorescein, angiograms were obtained at 0-1 minute (early phase) and 8-9 minutes (late phase) after injection. Grade of CNV was estimated by a blinded observer that examined fluorescein leakage on the angiograms, according to the following criteria: grade 0 (no fluorescein leakage), grade 1 (very mild leakage or staining), and grade 2 (fluorescein leakage indicating CNV).

### 2.13. Evaluation of Choroidal Neovascularization on Flat-Mounts

Eyes were enucleated at 2 and 8 weeks and fixed in 2% paraformaldehyde for 4 hours at room temperature. The RPE-choroid-sclera complex was isolated and, after permeabilization with 1% Triton X-100 in PBS for 1 hour at room temperature, incubated at 4°C with FITC-conjugated isolectin B4 (1 : 50; Sigma-Aldrich), which labels endothelial cells. After overnight incubation, the RPE-choroid-sclera complex was washed with PBS and mounted on glass slides, using Mowiol (Carl Roth GmbH, Karlsruhe, Germany), examined by fluorescence microscopy, photographed, and analyzed with ImageJ software.

### 2.14. Statistical Analysis

Statistical analysis of all experiments was performed using one-way ANOVA with Dunnett's multiple comparison test (GraphPad Prism 5; GraphPad Software, La Jolla, CA).

## 3. Results

### 3.1. PEDF Sequence Homology

Sequence analysis of the recombinant PEDF showed an 87.7% and 80.9% homology with bovine and rat PEDF, respectively. The two functional epitopes, which are responsible for the antiangiogenic and neurotrophic activity of PEDF [50], showed a 93.6% homology with bovine PEDF and an 84.6% homology with rat PEDF, indicating that the recombinant human PEDF used in our studies would bind to the PEDF receptor on human as well as bovine and rat cells.

### 3.2. PEDF Secretion by Immortalized Rat RPE Cells

**Figure 1:**
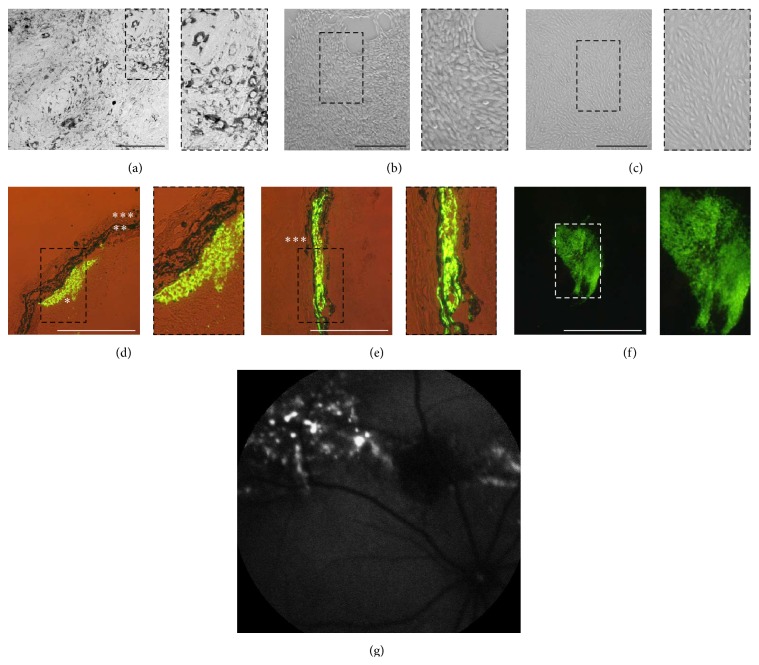
Morphology and localization of immortalized rat RPE cells. Primary rat RPE cells (a) were immortalized by transfection with pSV3-neo plasmid DNA.* In vitro* immortalized cells (b) were no longer pigmented but exhibited the same morphology as ARPE-19 cells (c). Cryosections of rat retinas at one day after transplantation (d) showed that the transplanted fluorescent cells transfected with the pT2-CAGGS-Venus transposon plasmid were localized adjacent to the RPE layer; by one week after transplantation (e) the cells appeared to have migrated away from the transplantation site (transfected cells^*∗*^, RPE^*∗∗*^, and choroid^*∗∗∗*^). A retinal flat-mount preparation (f) shows a cluster of transplanted fluorescent cells one week after transplantation. Fundus angiography illustrates the transplanted cells (fluorescence) at 3 months (g) after subretinal transplantation (scale bars: 200 *μ*m for (a); 500 *μ*m for (b)–(f); 200 *μ*m for (g); selected areas of (a)–(f) are 2x the original magnification).

**Figure 2:**
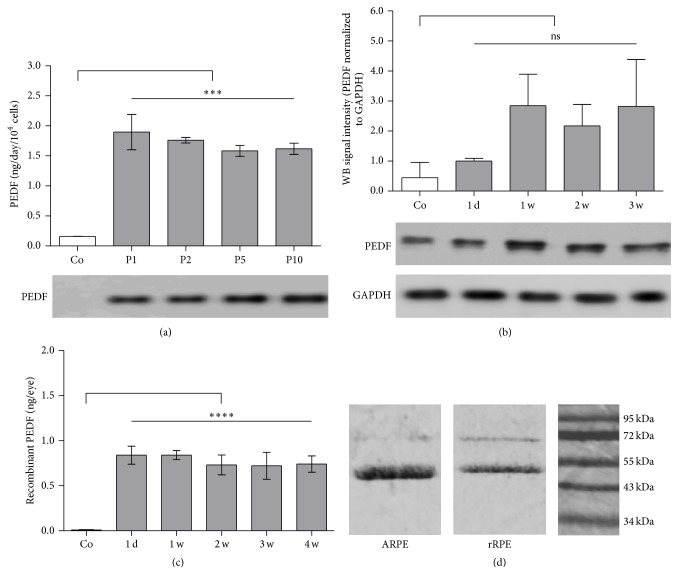
PEDF secretion by immortalized RPE cells* in vitro* and* in vivo*. (a) ELISA-based quantification and* Western* blot analysis of total PEDF secretion by immortalized rat RPE cells* in vitro* at passages 1, 2, 5, and 10 (grey bars) after* SB100X*-mediated transfection with the pT2-CMV-PEDF/EGFP transposon plasmid. Note that nontransfected control cells secreted only very small amounts of endogenous PEDF (white bar). Data represent the mean ± SD from 2 independent measurements (^*∗∗∗*^ *p* = 0.0001). (b)* Western* blot analysis of total PEDF extracted from rat control eyes and eyes at 1 day, 1, 2, and 3 weeks after subretinal transplantation of PEDF-transfected rat RPE cells. Double bands indicated the slightly different molecular weights of endogenous (46.5 kDa) and recombinant human PEDF (47.5 kDa), which included a His-tag. Loading of equal protein amounts was confirmed by similar densities of GAPDH protein bands (36 kDa). Note that for these experiments the number of cells transplanted was 1 × 10^5^, since the low sensitivity of the anti-PEDF antibodies required a larger amount of protein. Data represent the mean ± SD from 2 independent measurements. (c) ELISA-based quantification of recombinant PEDF secretion* in vivo* in lysates of rat eyes at 1 day, 1, 2, 3, and 4 weeks after subretinal transplantation of 1 × 10^4^ PEDF-transfected RPE cells, showed a constant level of recombinant human PEDF. For nontransfected control cells recombinant PEDF was not detectable (left bar), because the analysis was carried out with Ni-NTA HisSorb plates. Each bar represents the average data of three injected eyes (^*∗∗∗∗*^ *p* < 0.0001). (d) Coomassie blue-stained SDS-PAGE showed the Ni-NTA purified recombinant human PEDF, secreted by transfected ARPE-19 or immortalized rat RPE cells. Noncomplete saturation of the Ni-NTA bindings sites by rPEDF led to the copurification of a small amount of bovine serum albumin (light band at ~60 kDa), a component of the cell culture medium.

Isolated rat RPE cells exhibited the typical cobblestone morphology and pigmentation (Figure 1(a)). Immortalized rat RPE cells, obtained by transfection of primary cells with the pSV3-neo plasmid, were no longer pigmented but showed the same epithelial morphology as ARPE-19 cells (Figures 1(b) and 1(c)). The immortalized rat RPE cells became easily transfected and survived after transplantation to the subretinal space of rats for the 3 months the cells have been observed (Figures 1(d)–1(g)). Immortalized rat RPE cells, cotransfected with* SB100X* transposase and pT2-CMV-PEDF/EGFP transposon plasmids, expressed and secreted recombinant human PEDF. To obtain cells that secreted identical levels of PEDF, single transfected cells were selected and expanded by further cultivation. The clone which was used for further experiments secreted 2 ng PEDF/10^4^ cells/day, as analyzed by ELISA and immunoblotting (Figure 2(a)); the highest secretion level was reached 1 week after transfection and remained constant throughout the 10 passages the cells have been followed in culture.

### 3.3. Quantification of rPEDF in Rat Eyes after Transplantation

Based on the level of PEDF secreted by the transfected rat RPE cells, we estimated the number of cells necessary to achieve an optimal therapeutic effect when transplanted subretinally in a rat model of choroidal neovascularization. The level of total PEDF was determined by* Western* blot analysis in rat eyes transplanted with PEDF-transfected rat RPE cells at day 1, at 1, 2, and 3 weeks after subretinal transplantation. Compared to control eyes, a 2-fold increase in total PEDF was reached by the first week and remained constant during the 3 weeks of follow-up (Figure 2(b)). The amount of recombinant PEDF per transplanted rat eye remained constant at 0.72–0.84 ng during the 4 weeks of follow-up, as determined by ELISA using Ni-NTA HisSorb plates (Figure 2(c)). Recombinant human PEDF was purified with Ni-NTA resin from conditioned medium of either PEDF-transfected ARPE-19 or PEDF-transfected rat RPE cells (Figure 2(d)).

### 3.4. Neurite Outgrowth and Reduced Photoreceptor Degeneration

**Figure 3:**
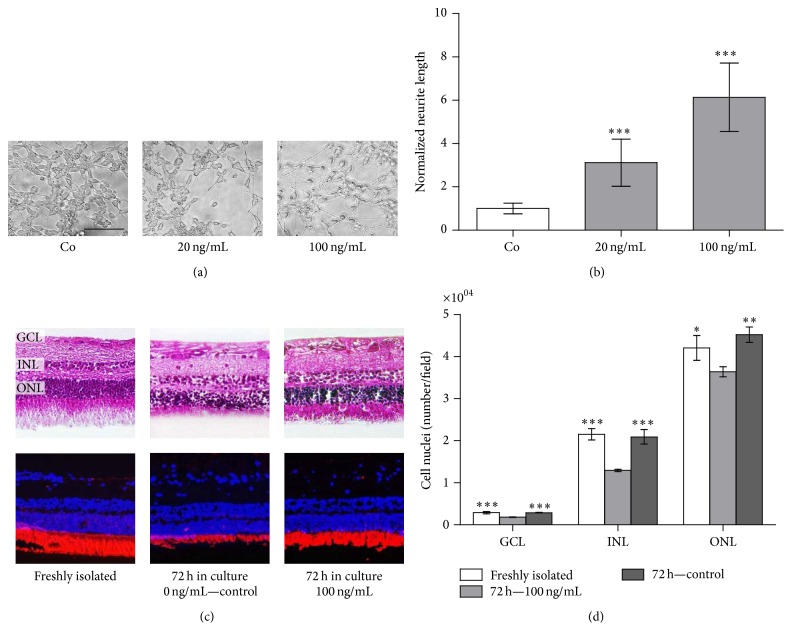
Neurogenic activity of purified recombinant PEDF. (a) 5 days after the addition of 20 and 100 ng/mL of rPEDF, it was evident that rPEDF stimulated neurite outgrowth (scale bar: 50 *μ*m). (b) Quantification showed a 3-fold increase of neurite outgrowth in the presence of 20 ng/mL rPEDF and a 6-fold increase in the presence of 100 ng/mL rPEDF. Data represents the mean ± SD of measurements from 6 microscopic fields from 2 independent experiments (^*∗∗∗∗*^ *p* < 0.0001; one-way ANOVA with Dunnett's multiple comparison test; control versus 20 and 100 ng/mL rPEDF). Values were normalized to the mean value of the control cultures. (c) Cultivated retinas in the presence of 0 and 100 ng/mL purified rPEDF showed that in the presence of rPEDF the loss of photoreceptors, as visualized with anti-rhodopsin antibodies (red), was significantly less than in control cultures. (d) Quantification of cell nuclei, visualized by staining with DAPI, in the ganglion cell layer (GCL) and the inner (INL) and the outer (ONL) nuclear layer, showed that the number of cells was the same in freshly isolated retinas and retinas treated with rPEDF. Data are presented as mean ± SD of the average number from 3 different sections. Statistical analysis showed significant differences between control and rPEDF-treated retinas as well as between control and freshly isolated retinas in the GCL (^*∗∗∗*^ *p* = 0.0002), the INL (^*∗∗∗*^ *p* = 0.0003), and the ONL (^*∗∗*^ *p* = 0.0063).

Purified recombinant PEDF induced neurite outgrowth in the SH-SY5Y human neuroblastoma cell line forming well-branched structures (Figure 3(a)). Compared to nontreated controls, the neurite length, normalized to the area of the SH-SY5Y cell bodies, increased by 3.12 ± 1.09- and 6.14 ± 1.58-fold in the presence of 20 and 100 ng/mL rPEDF, respectively (Figure 3(b)). In organotypic cultures of bovine neural retinas, addition of recombinant PEDF protected the photoreceptor cell outer segments from degeneration, as visualized by hematoxylin/eosin and immunohistochemical staining (Figure 3(c)). At 72 hours after cultivation, the number of nuclei in the ganglion cell layer (GCL), the inner nuclear layer (INL), and the outer nuclear layer (ONL) was reduced to 63.3%, 60.0%, and 86.5%, respectively. However, supplementation with 100 ng/mL rPEDF protected the cell nuclei in all layers with only a decrease of 1.10% in the GCL and 2.92% in the INL compared to freshly isolated retinas; the number of cell nuclei in the ONL was slightly increased (Figure 3(d)).

### 3.5. Inhibition of Angiogenic HUVEC Sprouting by Purified rPEDF

**Figure 4:**
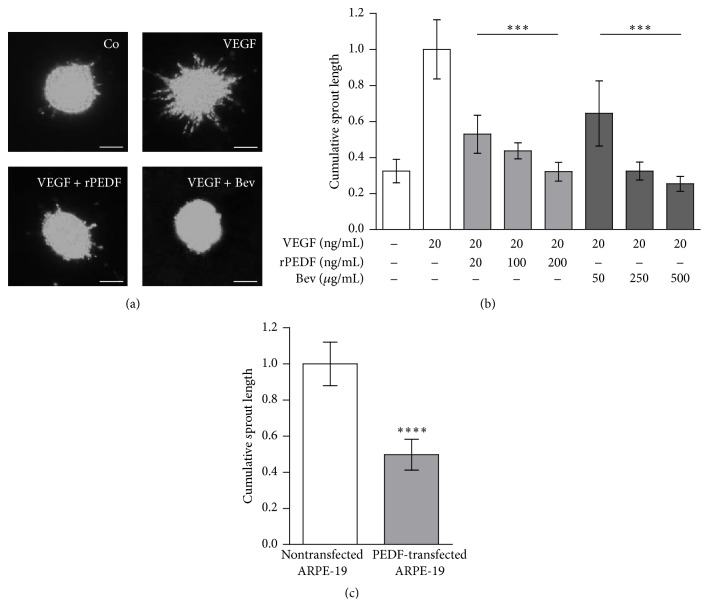
Inhibition of HUVEC sprouting by recombinant PEDF. (a) Exemplary fluorescence micrographs of DiO labeled HUVEC spheroids cultured in the presence of 2% serum, VEGF, VEGF plus rPEDF, and VEGF plus bevacizumab (scale bars: 50 *μ*m). (b) Effect of rPEDF and bevacizumab on VEGF-stimulated sprouting of human umbilical vein endothelial cells (HUVEC) spheroids. VEGF increased the cumulative sprouting length by more than 60% above control. The addition of rPEDF at a concentration of 200 ng/mL reduced sprouting below the control level. To achieve the same level of inhibition as 200 ng/mL of rPEDF, it required 250 *μ*g/mL of bevacizumab. The differences between VEGF-induced HUVEC sprouting and all concentrations of rPEDF and bevacizumab were statistically significant (^*∗∗∗∗*^ *p* < 0.0001; one-way ANOVA with Dunnett's multiple comparison test; VEGF versus VEGF + recombinant PEDF and VEGF + bevacizumab). The mean cumulative sprout length per spheroid was calculated by measuring and averaging the length of all sprouts from 10 randomly selected spheroids. (c) Cultures of HUVEC spheroids in the presence of 150,000 nontransfected ARPE-19 cells and 20 ng/mL VEGF (white bar) showed significant sprouting, whereas, in the presence of 150,000 transfected ARPE-19 cells producing 30 ng rPEDF/day (grey bar), sprouting was reduced by 50% (^*∗∗∗∗*^ *p* < 0.0001).

The addition of VEGF to HUVEC spheroids elicited the formation of sprouts, which was inhibited by recombinant PEDF and bevacizumab (Figure 4(a)). Compared to control, 20 ng/mL of VEGF increased cumulative sprout length 3.1 times. Sprouting was reduced by the anti-VEGF monoclonal antibody bevacizumab by 36%, 68%, and 75% at concentrations of 50, 250, and 500 *μ*g/mL, respectively. The addition of purified recombinant PEDF reduced VEGF-induced sprouting by 47%, 56%, and 68% at concentrations of 20, 100, and 200 ng/mL, respectively (Figure 4(b)). The cocultivation of spheroids with PEDF-transfected ARPE-19 cells resulted in the inhibition of HUVEC sprouting by 50%, compared to spheroids cultivated in the presence of nontransfected ARPE-19 cells (Figure 4(c)).

### 3.6. Enhanced Apoptosis by Purified rPEDF

**Figure 5:**
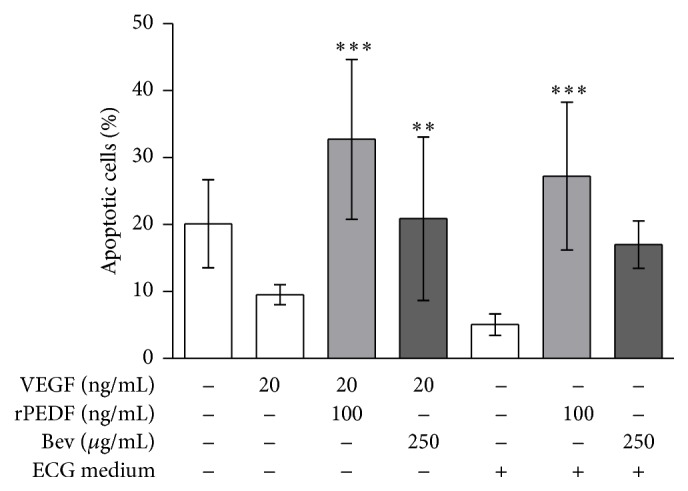
Effect of recombinant PEDF on apoptosis of HUVEC. The addition of VEGF protected HUVEC from apoptosis induced by low serum. Apoptosis of HUVEC was significantly increased by the addition of rPEDF or bevacizumab (^*∗∗∗∗*^ *p* < 0.0001; one-way ANOVA with Dunnett's multiple comparison test; VEGF/ECG medium versus VEGF/ECG medium + rPEDF and bevacizumab). Data are presented as mean ± SD of the average apoptotic cell number from at least 6 micrographs.

In medium containing 0.2% fetal bovine serum (FBS), 20.1% of HUVEC was apoptotic. Supplementation with 20 ng/mL of VEGF reduced the number of apoptotic cells to 9.53%, whereas the addition of 100 ng/mL rPEDF or 250 *μ*g/*μ*L bevacizumab increased apoptosis to 32.7% and 20.9%, respectively (Figure 5). In endothelial cell growth (ECG) medium containing 10% FBS, only 5.05% of HUVEC was apoptotic and the addition of 100 ng/mL rPEDF and 250 *μ*g/mL bevacizumab increased the number of apoptotic cells to 27.2% and 17.0%, respectively (Figure 5).

### 3.7. Inhibition of Cell Migration by rPEDF

**Figure 6:**
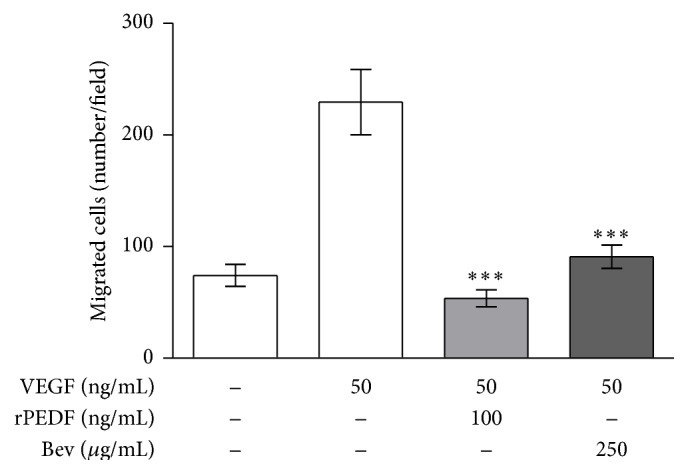
Effect of recombinant PEDF on migration of HUVEC. Quantitative analysis showed that migration of HUVEC was stimulated by VEGF and that VEGF-stimulated migration was significantly inhibited by rPEDF and bevacizumab (^*∗∗∗∗*^ *p* < 0.0001; one-way ANOVA with Dunnett's multiple comparison test; VEGF versus VEGF + rPEDF and bevacizumab). Cells of 10 randomly selected microscopic fields were enumerated and presented as mean ± SD.

Migration of endothelial cells is an essential step in the process of blood vessel growth and VEGF acts as a potent activator of HUVEC migration. Spontaneous migration by HUVEC was low, 74.2 ± 9.82 cells/field migrated across the filter. However, in the presence of 50 ng/mL VEGF the number of migrated cells increased to 229.6 ± 29.22 cells/field, whereas the addition of 100 ng/mL recombinant PEDF and 250 *μ*g/mL bevacizumab reduced VEGF-stimulated HUVEC migration to 53.6 ± 7.53 and 90.8 ± 10.5 cells/field, respectively (Figure 6).

### 3.8. Effect of rPEDF Secreted by Transfected Rat RPE Cells on Laser-Induced CNV Lesions

**Figure 7:**
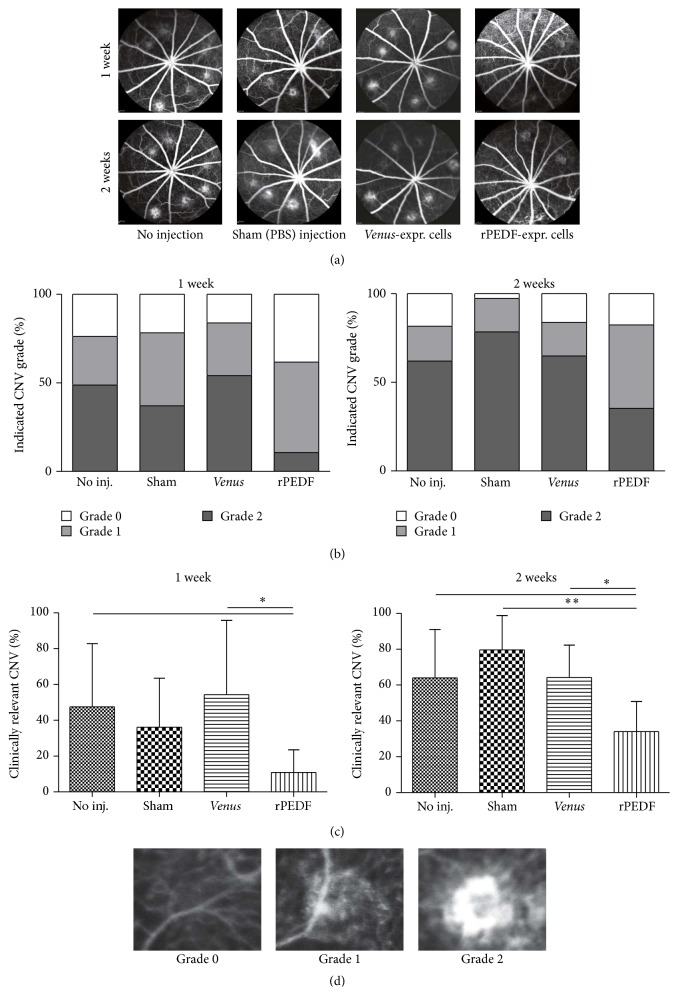
Effects of subretinal transplantation of RPE cells transfected with the* PEDF* gene on laser-induced CNV in a rat model. (a) Fluorescein angiograms of rat eyes 1 and 2 weeks after laser injury and cell transplantation showed the decrease in leakage in eyes transplanted with transfected RPE cells secreting 2 ng rPEDF/day, but not in eyes transplanted with* Venus*-expressing cells or sham-operated eyes. (b) Distribution of total CNV lesions, classified by a blinded observer into grade 0 (no leakage), grade 1 (very mild leakage), and grade 2 (clear leakage), showed that the eyes transplanted with PEDF-transfected cells had fewer grade 2 lesions than the other groups. Each group comprised at least 6 animals. (c) Quantification of clinically relevant grade 2 CNV lesions in rat eyes after 1 and 2 weeks showed that eyes transplanted with rPEDF-expressing cells had significantly fewer grade 2 lesions (1 week: ^*∗*^ *p* = 0.042; 2 weeks: ^*∗∗*^ *p* = 0.0023). (d) Exemplary fluorescein angiograms of grades 0, 1, and 2 lesions.

Fluorescein angiography was carried out to analyze the grade of CNV leakage in rat eyes transplanted subretinally with 1 × 10^4^ PEDF-transfected rat RPE cells, secreting approximately 2 ng PEDF/day, as well as in eyes transplanted with* Venus*-expressing cells, noninjected eyes, and sham-injected eyes. Representative angiograms were taken 1 and 2 weeks after laser treatment (Figure 7(a)). In rat eyes transplanted with PEDF-transfected rat RPE cells, lesion development clearly showed reduced fluorescein leakage at both 1 and 2 weeks after laser treatment. At days 7 and 14 after laser treatment, the incidence of grades 0, 1, and 2 lesions was similar in noninjected eyes and eyes transplanted with* Venus*-transfected cells. In sham-injected eyes, grade 2 lesions were reduced by 24.3% and grade 1 lesions increased by 50.8% at day 7 after laser treatment; after 14 days, the number of grade 2 lesions increased slightly to 26.5%, whereas the number of grade 1 lesions did not change. In PEDF-treated eyes, a reduction in grade 2 lesions of 78.2% and 43.1% was observed after 7 and 14 days, respectively (Figure 7(b)). Detailed statistical analysis of the clinically relevant grade 2 lesions revealed that the differences between noninjected, sham-injected, and control-transplanted eyes and eyes transplanted with PEDF-transfected cells were significant both at week 1 (^*∗*^ *p* = 0.042) and at week 2 (^*∗∗*^ *p* = 0.0023) (Figure 7(c)).

### 3.9. rPEDF-Mediated Reduction of CNV Area Measured on Choroidal Flat-Mounts

**Figure 8:**
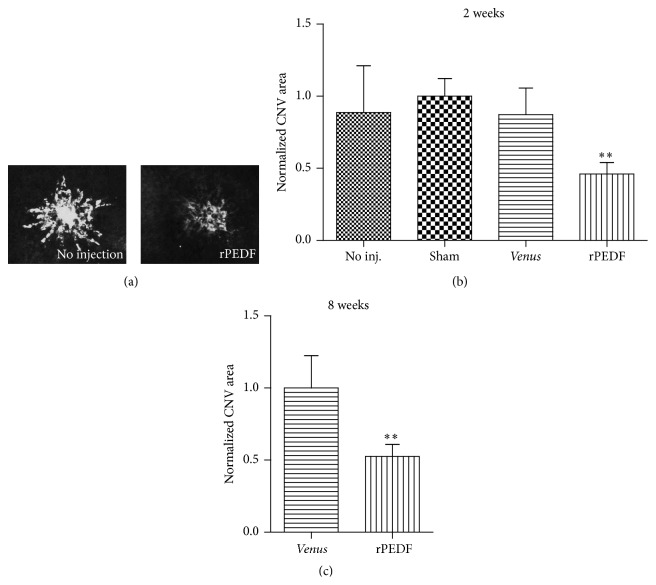
Choroidal flat-mounts of retinas from a rat model of laser-induced CNV. (a) Exemplary fluorescence micrographs of isolectin B4 (vascular leakage) stained choroidal flat-mounts 2 weeks after laser treatment. Note the decrease in B4 staining in the retinas transplanted with RPE cells transfected with the* PEDF* gene secreting 2 ng rPEDF/day. (b) Quantitative analysis of area of vascular leakage 2 weeks after laser injury showed that transplantation of PEDF-transfected RPE cells reduced the area of leakage by over 50% compared to control, sham-operated eyes, and eyes transplanted with* Venus*-expressing cells (^*∗∗*^ *p* = 0.0012, rPEDF versus no injection, sham injection, and* Venus*-expressing cells). (c) Choroidal flat-mounts of rat eyes 8 weeks after laser injury showed that the reduction in leakage observed at 2 weeks in eyes transplanted with PEDF-transfected RPE cells was still approximately 50% of the leakage in eyes transplanted with* Venus*-expressing cells (^*∗∗*^ *p* = 0.0022).

Analysis of isolectin B4 stained choroidal flat-mounts, prepared 2 weeks after laser treatment and subretinal transplantation of PEDF-transfected rat RPE cells, showed that the CNV lesion area was reduced compared to noninjected control eyes (Figure 8(a)). The area of CNV in eyes transplanted with PEDF-transfected rat RPE cells was 46.1% of the area of CNV in eyes injected with PBS; the area of CNV in noninjected eyes and eyes transplanted with* Venus*-expressing cells was 88.8% and 87.2% of the area of PBS-injected eyes, respectively. Statistical analysis showed that the differences between eyes transplanted with PEDF-transfected rat RPE cells and all other groups were significant, with a *p* value of less than 0.05 (Figure 8(b)). A comparison of the CNV area at 8 weeks after laser treatment showed a reduction of 47.5% in eyes transplanted with PEDF-transfected rat RPE cells compared to eyes transplanted with* Venus*-transfected cells (Figure 8(c)).

## 4. Discussion

The retinal pigment epithelium maintains the avascularity of the retina and a healthy choroidal vasculature by expressing and maintaining a balance between angiogenic and antiangiogenic factors, in particular VEGF and PEDF. The evidence that in neovascular AMD the equilibrium is shifted in favor of the angiogenic VEGF [51, 52] led to the development of inhibitors of VEGF, for example, the humanized monoclonal antibody bevacizumab (Avastin), its Fab fragment ranibizumab (Lucentis), and aflibercept (Eylea), which engendered a new era for the treatment of neovascular AMD and other ocular neovascular diseases [53–55]. We theorized that the side effects, resulting from frequent intravitreal injections of anti-VEGF antibodies to prevent CNV, could be alleviated by subretinal transplantation of genetically modified pigment epithelial cells that continuously secrete antiangiogenic PEDF. Autologous pigment epithelial cells transplanted to the subretinal space in neovascular AMD patients [28, 29] were well tolerated over a long period of time. However, no significant beneficial effects have been shown on vision. We postulate that vision improvement requires the inhibition of neovascularization by higher levels of PEDF. To insure that the transplanted cells secrete continuously a sufficient quantity of recombinant PEDF, we have developed an efficient nonviral transfection protocol, based on the* Sleeping Beauty* (*SB100X*) transposon system, and have shown that recombinant PEDF secretion in culture by PEDF-transfected cells was stable and constant for more than 1 year [41].

PEDF is a multifunctional protein that exhibits varied cellular actions, indicating that there are distinctive receptors that elicit diverse events. In fact, the two functional epitopes identified, a 34-mer peptide (residues 24–57) and a 44-mer peptide (residues 58–101) [50], appear to bind to two different receptors: the 44-mer peptide binds to a 80 kDa protein found in retinoblastoma Y-79 cells, neuronal cells, and retinal cells [56, 57] and induces neuroblastoma cell differentiation but does not prevent vascular leakage, whereas the 34-mer peptide binds to a 60 kDa receptor on endothelial cells [58] and prevents vascular leakage, suppresses new but not established blood vessels, and has no effect on retinoblastoma cells. Since the ultimate goal of transplanting PEDF-transfected pigment epithelial cells subretinally is for the cells to secrete active PEDF to inhibit CNV, it is critical that the recombinant PEDF secreted by the transfected cells is fully functional. In this study, we analyzed the functionality of purified recombinant human PEDF secreted by ARPE-19 and rat RPE cells transfected with the* PEDF* gene using the* SB100X* transposon system. Affinity-purified recombinant PEDF showed neurotrophic activity, as evidenced by the differentiation of human neuroblastoma cells in culture and protection of photoreceptor cells from degeneration in organ cultures of neural retinas.* In vitro*, the antiangiogenic properties of recombinant PEDF were confirmed by the inhibition of VEGF-stimulated sprouting and chemotaxis: rPEDF, secreted by RPE cells transfected using the* SB100X* transposon, is a potent inhibitor of HUVEC sprouting, the inhibition is concentration dependent, and the inhibitory activity is approximately 2000 times more potent than the activity of bevacizumab.

The ultimate goal of transplanting pigment epithelial cells to the subretinal space is to populate the subretinal space with PEDF-transfected cells that continuously secrete rPEDF for the treatment of retinal degenerative diseases, such as neovascular AMD. Here we have shown that it is possible to inhibit CNV by transplanting pigment epithelial cells transfected with the* PEDF* gene that express constant elevated levels of PEDF, as evidenced by the suppression of the development of laser-induced CNV lesions by transplantation of PEDF-transfected rat RPE cells into the subretinal space of Long Evans rats. Rats transplanted with 1 × 10^4^ RPE cells secreting 2 ng rPEDF/day inhibited the development of clinically relevant grade 2 lesions by 78% and 43% after 1 and 2 weeks, respectively. Transplantation into the subretinal space of rats of 1 × 10^4^ rat RPE cells, expressing 2 ng rPEDF/day* in vitro*, resulted in a constant level of rPEDF of 0.72–0.84 ng rPEDF in the eye indicating that the subretinal environment supports the expression of the* PEDF* gene integrated into the RPE cell's genome. To our knowledge, this study indicates for the first time the concentration of PEDF that is necessary to suppress CNV* in vivo*. A study using virally transduced cells for transplantation in Royal College of Surgeons (RCS) rats analyzed the production of PEDF* in vitro* but not the amount actually present* in vivo* [59].

A critical consideration for the treatment of neovascular AMD with transplantation of PEDF-transfected cells is the amount of PEDF produced, since too little PEDF will have no effect and too much PEDF may result in receptor downregulation and an increase in neovascularization [60]. Our laboratory is now in the process of investigating the optimal level of secreted PEDF as well as its lower and upper limits that will have a significant effect* in vivo*.

## 5. Conclusions

The results demonstrate that* SB100X*-transfected pigment epithelial cells secrete constant levels of rPEDF. Homologous PEDF-transfected rat RPE cells transplanted to the subretinal space prevent development of severe CNV lesions and limit the area of neovascularization in a rat model of CNV. Since* SB100X*-mediated transfection integrates the transgene and since RPE cells survive in the subretinal space, autologous* SB100X*-mediated PEDF-transfected cells transplanted to the subretinal space of neovascular AMD patients should secrete recombinant PEDF and inhibit CNV for the life of the patients.

## References

[1] Kaur C., Sivakumar V., Foulds W. S., Luu C. D., Ling E.-A. (2012). Hypoxia-induced activation of N-methyl-D-aspartate receptors causes retinal ganglion cell death in the neonatal retina. *Journal of Neuropathology and Experimental Neurology*.

[2] Adamis A. P., Shima D. T., Yeo K.-T. (1993). Synthesis and secretion of vascular permeability factor/vascular endothelial growth factor by human retinal pigment epithelial cells. *Biochemical and Biophysical Research Communications*.

[3] Ferrara N., Gerber H.-P., LeCouter J. (2003). The biology of VEGF and its receptors. *Nature Medicine*.

[4] Bouck N. (2002). PEDF: anti-angiogenic guardian of ocular function. *Trends in Molecular Medicine*.

[5] Dawson D. W., Volpert O. V., Gillis P. (1999). Pigment epithelium-derived factor: a potent inhibitor of angiogenesis. *Science*.

[6] Farnoodian M., Kinter J. B., Yadranji Aghdam S., Zaitoun I., Sorenson C. M., Sheibani N. (2015). Expression of pigment epithelium-derived factor and thrombospondin-1 regulate proliferation and migration of retinal pigment epithelial cells. *Physiological Reports*.

[7] Parmeggiani F., Campa C., Costagliola C. (2010). Inflammatory mediators and angiogenic factors in choroidal neovascularization: pathogenetic interactions and therapeutic implications. *Mediators of Inflammation*.

[8] Bai Y., Zhao M., Zhang C. (2014). Anti-angiogenic effects of a mutant endostatin: a new prospect for treating retinal and choroidal neovascularization. *PLoS ONE*.

[9] Marneros A. G., She H., Zambarakji H. (2007). Endogenous endostatin inhibits choroidal neovascularization. *The FASEB Journal*.

[10] Uno K., Bhutto I. A., McLeod D. S., Merges C., Lutty G. A. (2006). Impaired expression of thrombospondin-1 in eyes with age related macular degeneration. *British Journal of Ophthalmology*.

[11] Huber M., Wachtlin J. (2012). Vitreous levels of proteins implicated in angiogenesis are modulated in patients with retinal or choroidal neovascularization. *Ophthalmologica*.

[12] Ohno-Matsui K., Morita I., Tombran-Tink J. (2001). Novel mechanism for age-related macular degeneration: an equilibrium shift between the angiogenesis factors VEGF and PEDF. *Journal of Cellular Physiology*.

[13] Bhutto I. A., McLeod D. S., Hasegawa T. (2006). Pigment epithelium-derived factor (PEDF) and vascular endothelial growth factor (VEGF) in aged human choroid and eyes with age-related macular degeneration. *Experimental Eye Research*.

[14] Mohan N., Monickaraj F., Balasubramanyam M., Rema M., Mohan V. (2012). Imbalanced levels of angiogenic and angiostatic factors in vitreous, plasma and postmortem retinal tissue of patients with proliferative diabetic retinopathy. *Journal of Diabetes and its Complications*.

[15] Kim S. Y., Mocanu C., Mcleod D. S. (2003). Expression of pigment epithelium-derived factor (PEDF) and vascular endothelial growth factor (VEGF) in sickle cell retina and choroid. *Experimental Eye Research*.

[16] Sonmez K., Drenser K. A., Capone A., Trese M. T. (2008). Vitreous levels of stromal cell-derived factor 1 and vascular endothelial growth factor in patients with retinopathy of prematurity. *Ophthalmology*.

[17] Vasudev N. S., Reynolds A. R. (2014). Anti-angiogenic therapy for cancer: current progress, unresolved questions and future directions. *Angiogenesis*.

[18] Wells J. A., Glassman A. R., Ayala A. R. (2015). Aflibercept, bevacizumab, or ranibizumab for diabetic macular edema. *The New England Journal of Medicine*.

[19] Zhou Y., Jiang Y., Bai Y., Wen J., Chen L. (2015). Vascular endothelial growth factor plasma levels before and after treatment of retinopathy of prematurity with ranibizumab. *Graefe's Archive for Clinical and Experimental Ophthalmology*.

[20] Martin D. F., Maguire M. G., Ying G.-S., Grunwald J. E., Fine S. L., Jaffe G. J. (2011). Ranibizumab and bevacizumab for neovascular age-related macular degeneration. *The New England Journal of Medicine*.

[21] Pinheiro-Costa J., Costa J. M., Beato J. N. (2015). Switch to aflibercept in the treatment of neovascular AMD: one-year results in clinical practice. *Ophthalmologica*.

[22] Zampros I., Praidou A., Brazitikos P., Ekonomidis P., Androudi S. (2012). Antivascular endothelial growth factor agents for neovascular age-related macular degeneration. *Journal of Ophthalmology*.

[23] Pieramici D. J., Avery R. L. (2006). Ranibizumab: treatment in patients with neovascular age-related macular degeneration. *Expert Opinion on Biological Therapy*.

[24] Rosenfeld P. J., Rich R. M., Lalwani G. A. (2006). Ranibizumab: phase III clinical trial results. *Ophthalmology Clinics of North America*.

[25] Adelman R. A., Zheng Q., Mayer H. R. (2010). Persistent ocular hypertension following intravitreal bevacizumab and ranibizumab injections. *Journal of Ocular Pharmacology and Therapeutics*.

[26] Krishnan R., Goverdhan S., Lochhead J. (2009). Submacular haemorrhage after intravitreal bevacizumab compared with intravitreal ranibizumab in large occult choroidal neovascularization. *Clinical and Experimental Ophthalmology*.

[27] Carneiro Â. M., Barthelmes D., Falcão M. S. (2011). Arterial thromboembolic events in patients with exudative age-related macular degeneration treated with intravitreal bevacizumab or ranibizumab. *Ophthalmologica*.

[28] Falkner-Radler C. I., Krebs I., Glittenberg C. (2011). Human retinal pigment epithelium (RPE) transplantation: outcome after autologous RPE-choroid sheet and RPE cell-suspension in a randomised clinical study. *British Journal of Ophthalmology*.

[29] Aisenbrey S., Lafaut B. A., Szurman P. (2006). Iris pigment epithelial translocation in the treatment of exudative macular degeneration: a 3-year follow-up. *Archives of Ophthalmology*.

[30] Amaral J., Becerra S. P. (2010). Effects of human recombinant PEDF protein and PEDF-derived peptide 34-mer on choroidal neovascularization. *Investigative Ophthalmology and Visual Science*.

[31] Mori K., Gehlbach P., Yamamoto S. (2002). AAV-mediated gene transfer of pigment epithelium-derived factor inhibits choroidal neovascularization. *Investigative Ophthalmology & Visual Science*.

[32] Saishin Y., Silva R. L., Saishin Y. (2005). Periocular gene transfer of pigment epithelium-derived factor inhibits choroidal neovascularization in a human-sized eye. *Human Gene Therapy*.

[33] Amaral J., Fariss R. N., Campos M. M. (2005). Transscleral-RPE permeability of PEDF and ovalbumin proteins: implications for subconjunctival protein delivery. *Investigative Ophthalmology & Visual Science*.

[34] Ginn S. L., Liao S. H., Dane A. P. (2010). Lymphomagenesis in SCID-X1 mice following lentivirus-mediated phenotype correction independent of insertional mutagenesis and *γ*c overexpression. *Molecular Therapy*.

[35] Hartman Z. C., Black E. P., Amalfitano A. (2007). Adenoviral infection induces a multi-faceted innate cellular immune response that is mediated by the toll-like receptor pathway in A549 cells. *Virology*.

[36] Nair V. (2008). Retrovirus-induced oncogenesis and safety of retroviral vectors. *Current Opinion in Molecular Therapeutics*.

[37] Maguire A. M., Simonelli F., Pierce E. A. (2008). Safety and efficacy of gene transfer for Leber's congenital amaurosis. *The New England Journal of Medicine*.

[38] Chévez-Barrios P., Chintagumpala M., Mieler W. (2005). Response of retinoblastoma with vitreous tumor seeding to adenovirus-mediated delivery of thymidine kinase followed by ganciclovir. *Journal of Clinical Oncology*.

[39] Ildefonso C. J., Kong L., Leen A. (2010). Absence of systemic immune response to adenovectors after intraocular administration to children with retinoblastoma. *Molecular Therapy*.

[40] Campochiaro P. A., Nguyen Q. D., Shah S. M. (2006). Adenoviral vector-delivered pigment epithelium-derived factor for neovascular age-related macular degeneration: results of a phase I clinical trial. *Human Gene Therapy*.

[41] Johnen S., Izsvák Z., Stöcker M. (2012). Sleeping beauty transposon-mediated transfection of retinal and iris pigment epithelial cells. *Investigative Ophthalmology & Visual Science*.

[42] Izsvák Z., Chuah M. K. L., VandenDriessche T., Ivics Z. (2009). Efficient stable gene transfer into human cells by the Sleeping Beauty transposon vectors. *Methods*.

[43] Izsvák Z., Hackett P. B., Cooper L. J. N., Ivics Z. (2010). Translating Sleeping Beauty transposition into cellular therapies: victories and challenges. *BioEssays*.

[44] Mátés L., Chuah M. K. L., Belay E. (2009). Molecular evolution of a novel hyperactive Sleeping Beauty transposase enables robust stable gene transfer in vertebrates. *Nature Genetics*.

[45] Yant S. R., Wu X., Huang Y., Garrison B., Burgess S. M., Kay M. A. (2005). High-resolution genome-wide mapping of transposon integration in mammals. *Molecular and Cellular Biology*.

[46] Korff T., Augustin H. G. (1998). Integration of endothelial cells in multicellular spheroids prevents apoptosis and induces differentiation. *Journal of Cell Biology*.

[47] Abramoff M. D., Garvin M. K., Sonka M. (2010). Retinal imaging and image analysis. *IEEE Reviews in Biomedical Engineering*.

[48] Kaempf S., Johnen S., Salz A. K., Weinberger A., Walter P., Thumann G. (2008). Effects of bevacizumab (Avastin) on retinal cells in organotypic culture. *Investigative Ophthalmology & Visual Science*.

[49] Kaempf S., Walter P., Salz A. K., Thumann G. (2008). Novel organotypic culture model of adult mammalian neurosensory retina in co-culture with retinal pigment epithelium. *Journal of Neuroscience Methods*.

[50] Filleur S., Volz K., Nelius T. (2005). Two functional epitopes of pigment epithelial-derived factor block angiogenesis and induce differentiation in prostate cancer. *Cancer Research*.

[51] Aiello L. P., Avery R. L., Arrigg P. G. (1994). Vascular endothelial growth factor in ocular fluid of patients with diabetic retinopathy and other retinal disorders. *The New England Journal of Medicine*.

[52] Kvanta A., Algvere P. V., Berglin L., Seregard S. (1996). Subfoveal fibrovascular membranes in age-related macular degeneration express vascular endothelial growth factor. *Investigative Ophthalmology & Visual Science*.

[53] Fong A. H. C., Lai T. Y. Y. (2013). Long-term effectiveness of ranibizumab for age-related macular degeneration and diabetic macular edema. *Clinical Interventions in Aging*.

[54] Palejwala N. V., Lauer A. K. (2014). Aflibercept: an update on recent milestones achieved. *Drugs of Today*.

[55] Stewart M. W. (2012). The expanding role of vascular endothelial growth factor inhibitors in ophthalmology. *Mayo Clinic Proceedings*.

[56] Alberdi E., Aymerich M. S., Becerra S. P. (1999). Binding of pigment epithelium-derived factor (PEDF) to retinoblastoma cells and cerebellar granule neurons. Evidence for a PEDF receptor. *The Journal of Biological Chemistry*.

[57] Aymerich M. S., Alberdi E. M., Martínez A., Becerra S. P. (2001). Evidence for pigment epithelium-derived factor receptors in the neural retina. *Investigative Ophthalmology & Visual Science*.

[58] Yamagishi S.-I., Inagaki Y., Nakamura K. (2004). igment epithelium-derived factor inhibits TNF-*α*-induced interleukin-6 expression in endothelial cells by suppressing NADPH oxidase-mediated reactive oxygen species generation. *Journal of Molecular and Cellular Cardiology*.

[59] Semkova I., Kreppel F., Welsandt G. (2002). Autologous transplantation of genetically modified iris pigment epithelial cells: a promising concept for the treatment of age-related macular degeneration and other disorders of the eye. *Proceedings of the National Academy of Sciences of the United States of America*.

[60] Apte R. S., Barreiro R. A., Duh E., Volpert O., Ferguson T. A. (2004). Stimulation of neovascularization by the anti-angiogenic factor PEDF. *Investigative Ophthalmology & Visual Science*.

